# Epithelioid and spindle rhabdomyosarcoma with TFCP2 rearrangement in abdominal wall: a distinctive entity with poor prognosis

**DOI:** 10.1186/s13000-023-01330-y

**Published:** 2023-03-30

**Authors:** Yuan Li, Dan Li, Jingyu Wang, Jinlong Tang

**Affiliations:** 1 Department of Pediatrics, the Children’s Hospital, Zhejiang University School of Medicine, National Clinical Research Center for Child Health, 310000 Hangzhou, Zhejiang China; 2 Department of Pathology, Ministry of scientific research and discipline construction, Affiliated Hospital of Jiaxing University, The First Hospital of Jiaxing, 314001 Jiaxing, Zhejiang China; 3 Department of Pathology, The Second Affiliated Hospital, Zhejiang University School of Medicine, 310009 Hangzhou, Zhejiang China

**Keywords:** Epithelioid and spindle rhabdomyosarcoma, TFCP2 rearrangement, Poor prognosis

## Abstract

**Background:**

Epithelioid and spindle rhabdomyosarcoma (ES-RMS) with TFCP2 rearrangement is a recently discovered rare variant of rhabdomyosarcoma composed of epithelioid and spindle cells, because it shows extraordinarily adverse prognosis and is easily misdiagnosed as other epithelioid or spindle cell tumors.

**Methods:**

A rare case of ES-RMS with TFCP2 rearrangement was presented and English literatures in Pubmed online up to 01 July 2022 were gathered by two authors for a systematic review according to the inclusion and exclusion criteria.

**Case presentation/results:**

We report a case of ES-RMS in an early 30s-years-old female, the neoplastic cells are remarkably immunoreactive with CK(AE1/AE3), and partially with ALK protein. Unexpectedly, the tumor shows TFCP2 rearrangement with coexistence of increased copy numbers of EWSR1 and ROS1 gene and MET gene mutation. Besides, Next-generation sequencing for genetic mutational profiling revealed frequent MET exon14 mutations in chromosome 7, most of which are C > T nonsynonymous SNV, and exon42 of ROS1 in chromosome 6 showed frequent G > T mutation up to 57.54%. In addition, neither MyoD1 mutation nor gene fusions were detected. Moreover, the patient shows high tumor mutational burden (TMB) up to 14.11 counts/Mb. Finally, as many cases of ES-RMS including our case had local progression or metastasis, we find, similar to epithelioid rhabdomyosarcoma (median survival time is 10 month), ES-RMS shows a more aggressive behavior and adverse prognosis (median survival time is 17 month) than spindle cell/sclerosing rhabdomyosarcoma (median survival time is 65 month) according previous studies.

**Conclusions:**

ES-RMS with TFCP2 rearrangement is a rare malignant tumor and easily confused with other epithelioid or spindle cell tumors, it may harbor additional gene alteration in addition to TFCP2 rearrangement, such as MET mutation, increased copy numbers of EWSR1 and ROS1 gene, high TMB. Most importantly, it may show very poor outcome with extensive metastasis.

**Supplementary Information:**

The online version contains supplementary material available at 10.1186/s13000-023-01330-y.

## Introduction

Rhabdomyosarcoma (RMS), a malignant tumor that extremely rarely occurs in both adults and pediatric patients, was divided into four major subtypes: pleomorphic, alveolar, spindle cell/sclerosing, embryonal, according the current WHO classification [1]. Nevertheless, as the advance of molecular biology technique, especially next-generation sequencing applying for the pathological diagnosis, numerous novel characteristic molecules involving neoplastic diagnosis have been unearthed and thus refined many tumor entities in mesenchymal tumor, such as HEY1-NCOA2 fusion in mesenchymal chondrosarcoma [2], EWSR1-NR4A3 fusion in extraskeletal myxoid chondrosarcoma [3], WWTR1-CAMAT1 fusion and YAP1-TFE3 fusion in epithelioid hemangioendothelioma [4, 5]. As regard to RMS, PAX3-FOXO1 and PAX7-FOX1 fusion were characteristics in alveolar RMS [6], and the MYOD1 mutation, EP300-VGLL3, NCOA2-MEIS1, CAV1-MET, YAP1-MAML2, EWSR1-UBP1 fusion in a small subset of spindle cell/sclerosing RMS (SS-RMS) [7–11]. Most importantly, rare cases of RMS characteristic by the profiling of epithelioid to spindle cells, namely epithelioid and spindle RMS (ES-RMS), were recently found to harbor EWSR1-TFCP2 or FUS-TFCP2 fusion and predominately arising in the bone of head and neck and pelvis, the patients with these RMS have extraordinarily adverse prognosis [12]. Here, we report a case of ES-RMS with TFCP2 rearrangement along with increased copy numbers of EWSR1 and ROS1 gene and MET gene mutation and show additional findings from next-generation sequencing, and the results from a systematic review showed ES-RMS with TFCP2 rearrangement had an inferior outcome.

## Methods

### Case report

Patient medical records were reviewed retrospectively at the Second Affiliated Hospital, Zhejiang University School of Medicine, and follow-up information was recorded by telephone and/or face-to face communication. Informed consent was obtained from the patient to future publish this article, and we assured that any data that was used would be anonymised. Furthermore, the research is approved by the human research ethical committee of the Second Affiliated Hospital, Zhejiang University School of Medicine.

### DNA and RNA next-generation sequencing analysis

For next-generation sequencing, a total DNA and RNA of paraffin embedded tissue were extracted respectively and then sent to be sequenced and analyzed according to Illumina’s protocols by Beijing Genetron Technology Co., Ltd (Beijing, China). All tissues were processed independently repeated three times. an Illumina HiSeq 3000 sequencer was used to sequence paired-end libraries. Briefly, mRNAs were selected using poly-T beads. Then, double stranded cDNAs were generated from RNAs fragmented and adaptors were ligated to be further sequenced. 5’ RACE PCR was employed for cDNA amplification. For samples close to the minimum input requirement, additional pre-capture PCR cycles were performed to generate sufficient PCR product for hybridization. RNA-seq and DNA-seq experiments have been performed on an Illumina HiSeq3000 using a paired-end read length of 2 × 150 pb with the Illumina HiSeq3000 Sequencing System (Illumina, San Diego, CA). Analysis on gene differential expression was performed with Cuffdiff in the Cufflinks package, gene differential expressions defined as q < 0.05 and |log2(fold change)| > 0.8 could further be analyzed and verified by quantitative PCR.

### Systematic literature review

A systematic review of the literature was conducted using Pubmed online according to the Preferred Reporting Items for Systematic reviews and Meta-Analyses (PRISMA) guideline [13], the keywords were below: ‘epithelioid and spindle rhabdomyosarcoma’ or ‘epithelioid rhabdomyosarcoma’ or ‘spindle rhabdomyosarcoma’ or ‘sclerosing rhabdomyosarcoma’ or ‘spindle cell/sclerosing rhabdomyosarcoma’ or ‘TFCP2 rearrangement’ and ‘rhabdomyosarcoma’. The search literatures were only restricted to English language up to 01 July 2022. Two authors reviewed published literatures. Literatures were gathered based on inclusion and exclusion criteria. The inclusion criteria were as follows: (1) literature with respect to epithelioid and spindle rhabdomyosarcoma with TFCP2 rearrangement, (2) literature involving epithelioid rhabdomyosarcoma, (3) literature mentions spindle cell/sclerosing rhabdomyosarcoma. The cases were excluded if other subtypes of rhabdomyosarcoma or malignant mesenchymal tumor were involved or rhabdomyosarcoma was only mentioned in general, we also excluded conference abstracts, commentaries, and opinions but examined their references for possible inclusions. Our main focuses were to review the overall clinicopathologic features and compare with prognosis significances within ES-RMS, E-RMS and SS-RMS, therefore, for each selected literature the following data were enrolled: age, sex, tumor size location, recurrence, metastasis, treatment, follow-up time and clinical outcome. Overall survival was accomplished using the Kaplan-Meier analysis by GraphPad Prism 8.0 (GraphPad, Software, San Diego, CA, US). *P* < 0.05 was considered as a statistical significance.

## Results

### Case report

#### Patient information

In Apirl, 2020, an early 30s-years-old female underwent the operation of excision of an abdominal wall mass about 5 × 4 × 3 cm due to a lump in the right abdominal wall for a month at the outside hospital. The patient has no history of any neoplasm diseases and family hereditary illnesses. According to the pathological diagnostic report from other hospital, myofibroblastoma was considered, and the immunohistochemical staining shows tumor cells were positive for SMA and Vimentin and negative for S100, CD68, CD34, EMA, P53, HMB45 and MART-1, the proliferative index Ki67 is 10%.

#### Clinical findings

Six months later, she presented with a recurrent lump at the same location of the abdominal wall for half a month and was admitted to the department of general surgery. Before operation, previous pathological sections were sent to consultation and the proliferative fasciitis was considered. On examination, she was found to have a firm, fixed skin mass without redness, ulceration, bleeding and overflow adjacent surgical scar in the right abdominal wall, and the Color Doppler Ultrasound showed a hypoechoic nodule about 0.57 × 0.52 × 0.64 cm in the subcutaneous soft tissue of the right abdominal wall with irregular shape, angular edge, uneven internal echo, increased echo of surrounding soft tissue, and no obvious blood flow signal is found in color doplor flow image (Supplementary Fig. 1). Therefore, the patient underwent excision of the abdominal wall mass and adjacent surgical scar again.

#### Diagnostic assessment

**Fig. 1:**
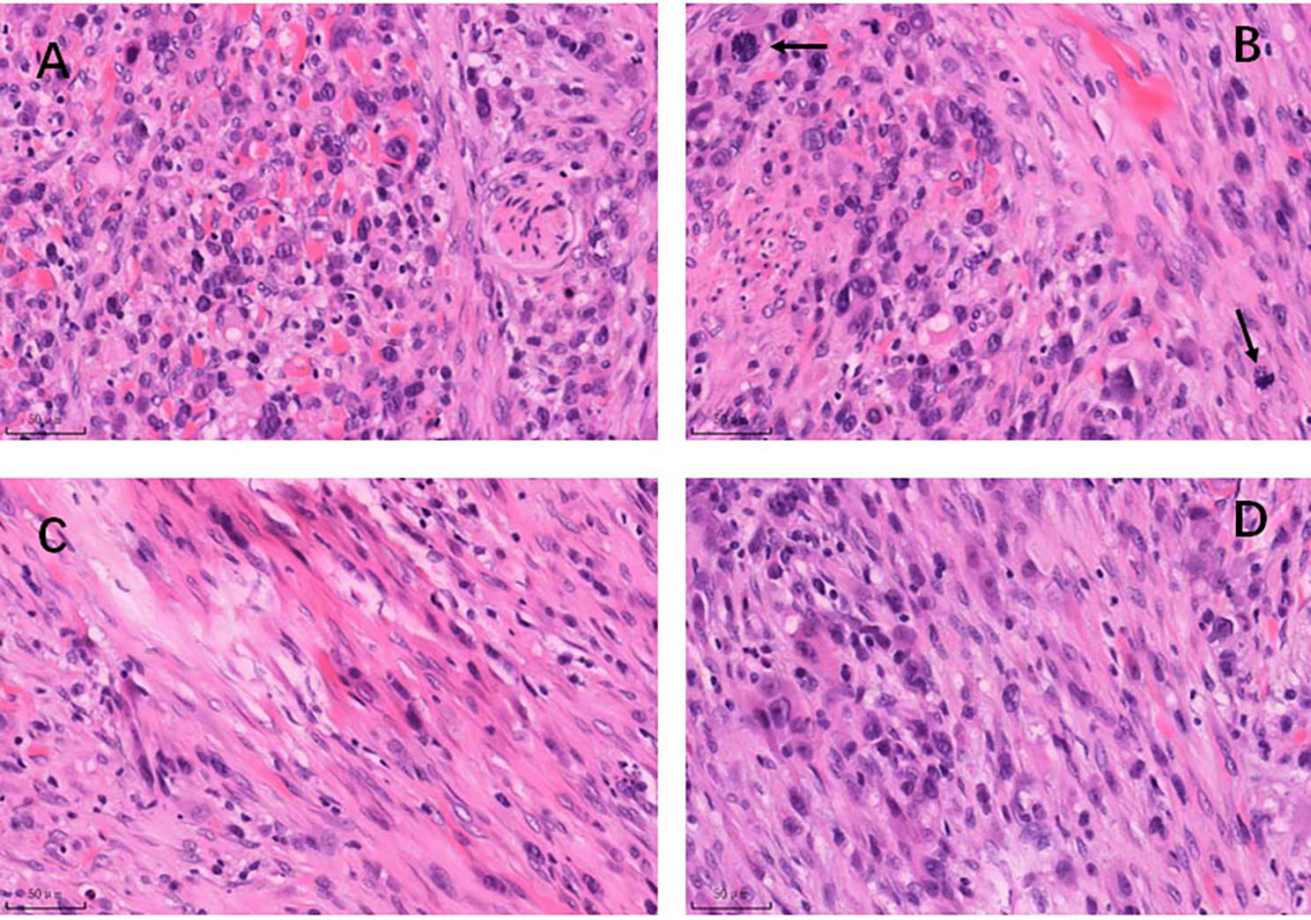
Microscopic findings of epithelioid and spindle cells rhabdomyosarcoma: Hematoxylin-eosin staining shows tumor is primarily composed of epithelioid cells and spindle cells, epithelioid cells show prominent cellular pleomorphism with obvious nucleoli, abundant and brilliant eosinophilic cytoplasm (Fig. 1A), and visible mitotic figures (Fig. 1B), spindle cells show hyperchromatic nucleus with prominent nucleoli and brilliant eosinophilic cytoplasm (Fig. 1C), two components can intertwine with each other in some regions (Fig. 1D). Bar: 50 μm, 200x

Grossly, the lesion is grey-white and red on cutting surface with obscure circumscription. Microscopic findings show a tumor with diffuse infiltration primarily composed of two cellular components: epithelioid cells and spindle cells, epithelioid cells show prominent cellular pleomorphism with obvious nucleoli, abundant and brilliant eosinophilic cytoplasm (Fig. 1A), and visible mitotic figures (Fig. 1B), spindle cells show hyperchromatic nucleus with prominent nucleoli and brilliant eosinophilic cytoplasm (Fig. 1C), two components intertwine with each other in some regions (Fig. 1D).

**Fig. 2:**
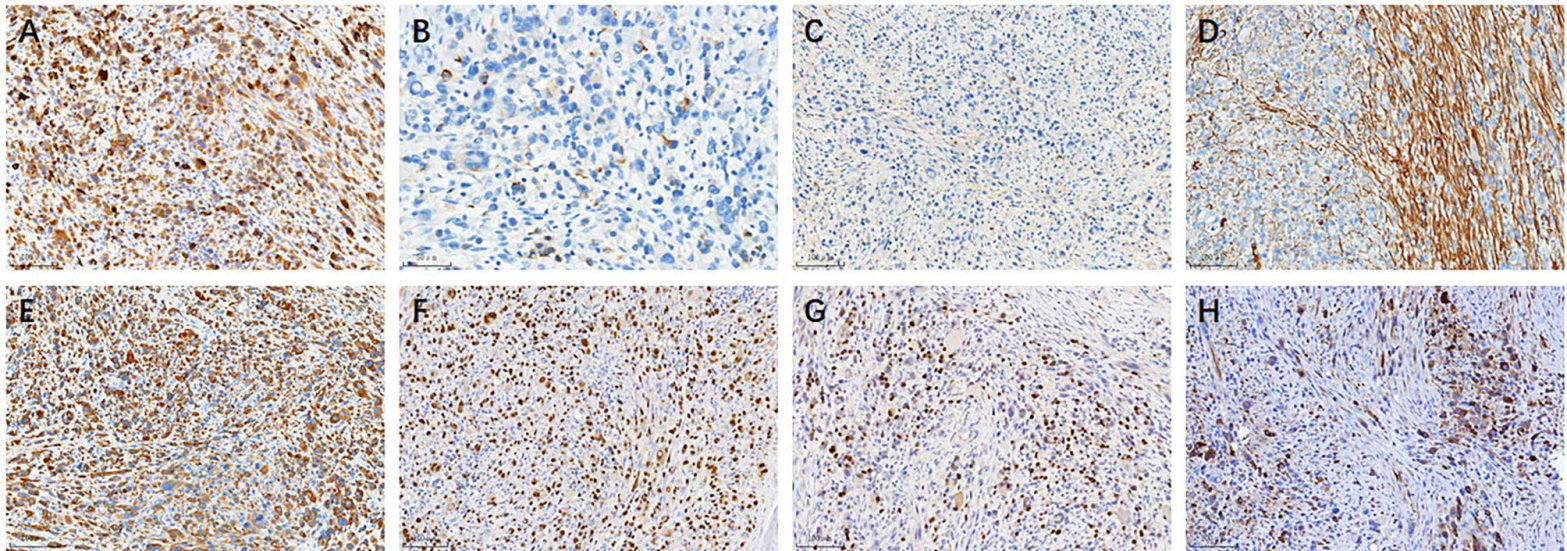
Immunohistochemical findings of epithelioid and spindle cells rhabdomyosarcoma: Neoplastic cells are remarkably immunoreactive with CK(AE1/AE3) (Fig. 2A), scattered with CK18 (Fig. 2B), but negative for EMA (Fig. 2C). SMA strongly express in spindle cells but partially in epithelioid cells (Fig. 2D). Noticeably, neoplastic cells strongly and diffusely express the skeletal muscle markers Desmin (Fig. 2E), MyoD1 (Fig. 2F) and Myogenin (Fig. 2G), and partially express ALK protein (Fig. 2H). Bar: 50 μm, 200x; 100 μm, 100x

**Fig. 3:**
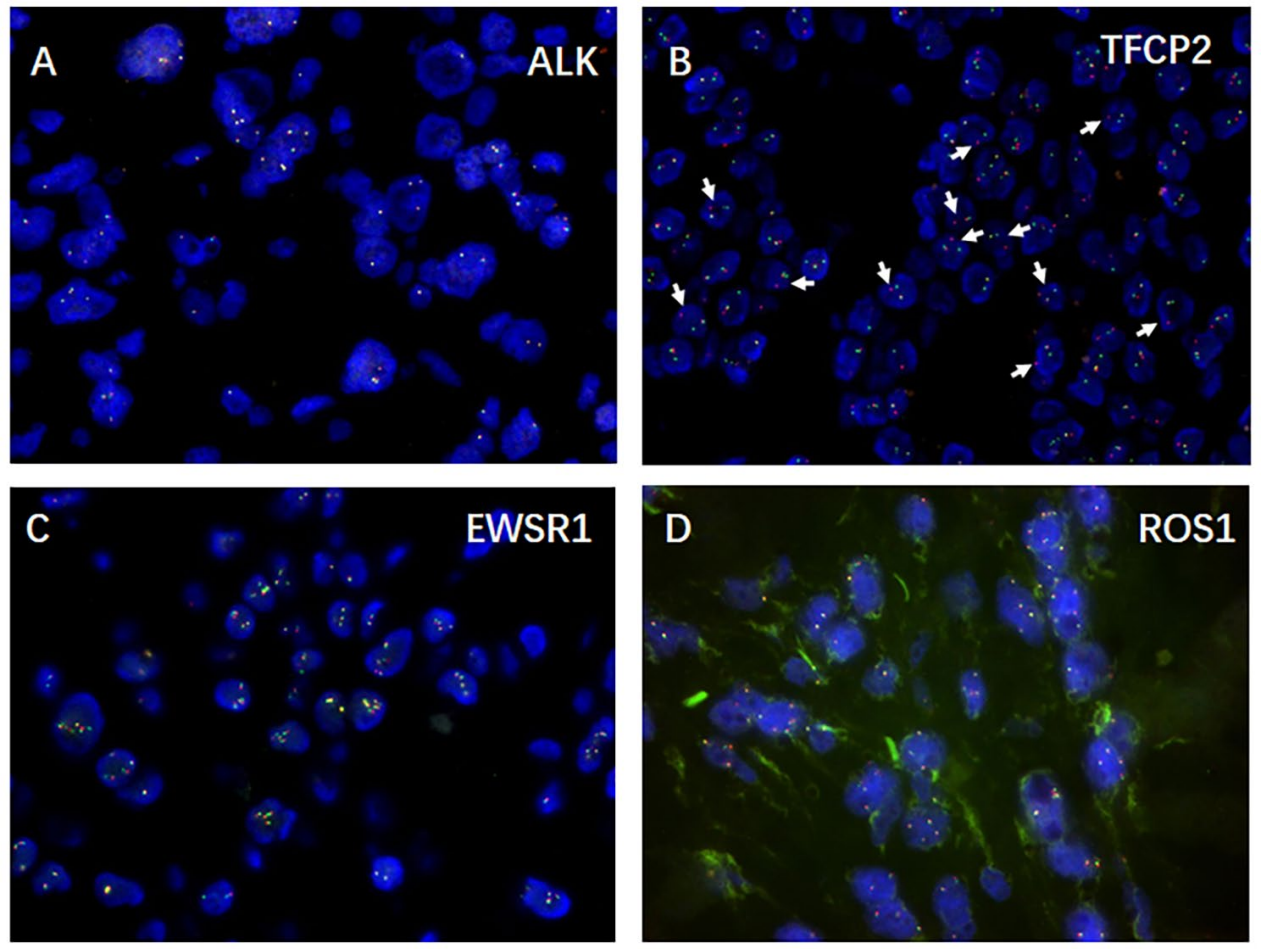
Molecular genetic findings of epithelioid and spindle cells rhabdomyosarcoma: Fluorescence in situ hybridization assay showed no ALK rearrangement (Fig. 3A) but rather TFCP2 rearrangement (Fig. 3B), the increased copy numbers of EWSR1 gene (Fig. 3C) and ROS gene (Fig. 3D).

Immunohistochemically, the neoplastic cells are remarkably immunoreactive with CK(AE1/AE3) (Fig. 2A), scattered with CK18 (Fig. 2B), but negative for EMA (Fig. 2C). SMA strongly expresses in spindle cells but partially in epithelioid cells (Fig. 2D). Most importantly, the neoplastic cells strongly and diffusely express the skeletal muscle markers Desmin (Fig. 2E), MyoD1 (Fig. 2F) and Myogenin (Fig. 2G). Unexpectedly, ALK protein is partially expressed in some neoplastic cells (Fig. 2H). In addition, it retains INI-1 expression and exhibits negativity for CD34. Moreover, the neoplastic cells are negative with CAM5.2, CK5/6, P40, CD31, ERG, CD30, S-100, HMB45, Melan-A. although, the neoplastic cells exhibit reactivity with ALK protein, Fluorescence in situ hybridization (FISH) assay showed no ALK rearrangement (Fig. 3A) or FUS fusion but rather TFCP2 rearrangement (Fig. 3B). Even more interesting is that the increased copy numbers of EWSR1 (Fig. 3C) and ROS1 gene (Fig. 3D) were as well detected in neoplastic cells. Therefore, a final diagnosis of ES-RMS with TCFCP2 rearrangement was rendered.

Next-generation sequencing for genetic mutational profiling revealed frequent MET exon14 mutations in chromosome 7, most of which are C > T nonsynonymous single nucleotide variation (Supplementary Table 1), and 15 somatically acquired mutations, including ROS1, SDHA, ADGRA2, CXCR4, LRP1B, XRCC3, ATIC, PIK3C2B, NSD2, SPTA1, PMS1, ASXL1, CCNE1, ARAF, TOP1, PLCG2 were as well detected. Of which, exon42 of ROS1 in chromosome 6 showed G > T, and frequency of mutation was up to 57.54% (Supplementary Table 2). In addition, neither MyoD1 mutation nor gene fusions were detected in the panel of sequencing genes. Moreover, the tumor tissues showed high tumor mutational burden (TMB) up to 14.11 counts/Mb according to the latest high TMB criterion defined as the top quartile or ≥ 2.80 counts/Mb [14].

#### Therapeutic intervention

After surgery, the patient underwent regular chemotherapy and traditional chinese medicine.

#### Follow-up and outcomes

The patient was found a mass in the right breast and humerus by regular examination of X-ray in the outside hospital until now, and metastatic neoplasm was considered.

### Systematic literature review

**Fig. 4:**
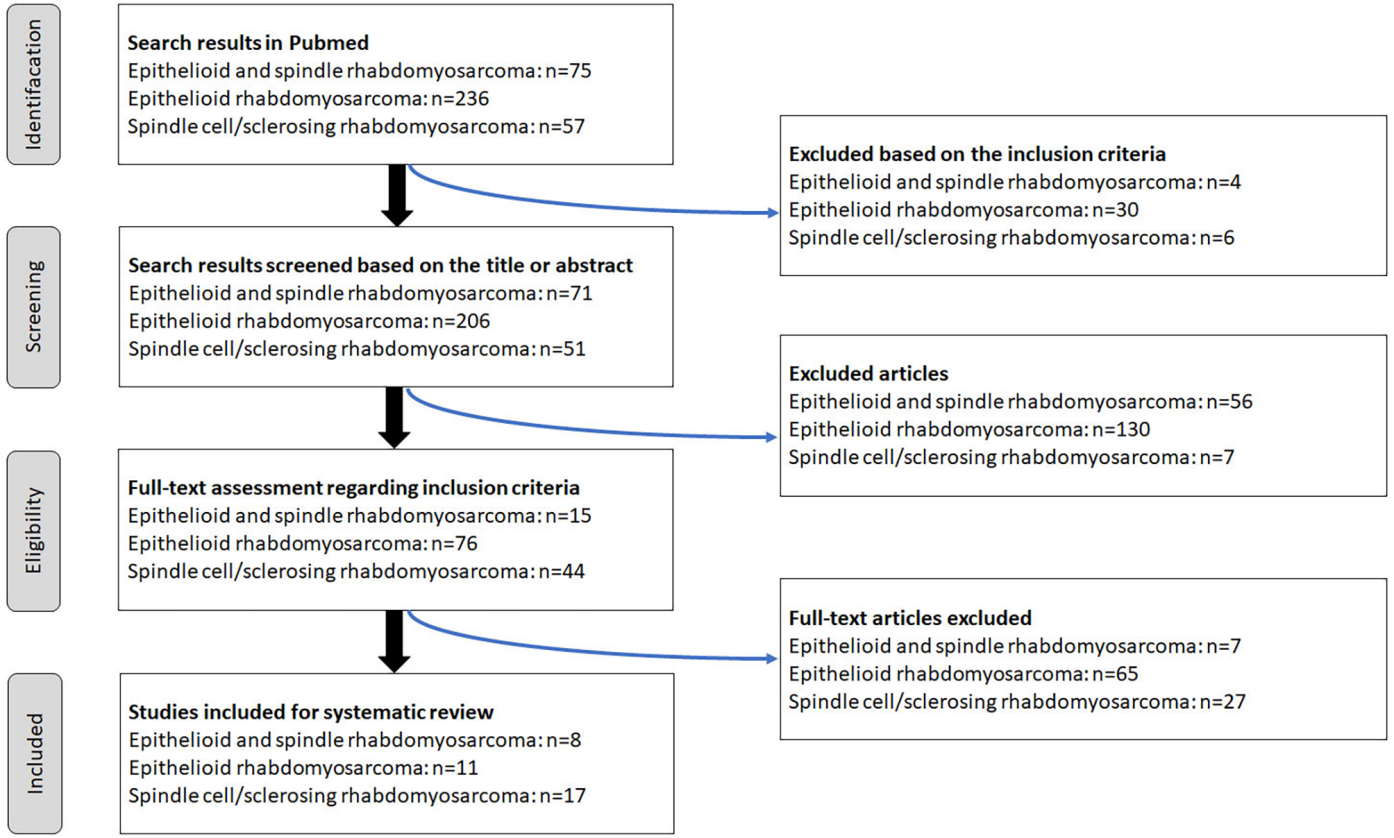
A PRISMA flowchart of selecting public articles involving ES-RMS, E-RMS and SS-RMS. ES-RMS: epithelioid and spindle rhabdomyosarcoma; E-RMS: epithelioid rhabdomyosarcoma; SS-RMS: spindle cell/sclerosing rhabdomyosarcoma

According to PRISMA guideline [13], a PRISMA flowchart of selecting public articles was detailly showed in Fig. 4. The search results identified 15 literatures involving ES-RMS, 76 literatures E-RMS, 44 SS-RMS, respectively. Of which, 7 literatures involving ES-RMS, 65 literatures E-RMS, 27 SS-RMS were excluded respectively due to other subtypes of rhabdomyosarcoma or malignant mesenchymal tumor were involved or rhabdomyosarcoma was only mentioned in general according to the excluded criteria. Finally, 8 literatures involving ES-RMS, 11 literatures E-RMS, 17 literatures SS-RMS were included, which is composed of 33 cases of ES-RMS including our case, 28 E-RMS, 71 SS-RMS, respectively.

According to systematic review, ES-RMS may occur from 11 to 86 years, and a median age is 27 years, and may predominately arise from head and neck, such as mandible, maxilla, and skull [12, 15–21] (Table 1), As ES-RMS shows partially morphologic overlap with epithelioid RMS (E-RMS) - a novel variant of RMS recently descripted by Jo et al. in 2011 [22] - and SS-RMS, we found that E-RMS, predominately occurs in elderly patients and has a median age about 55 years. Whereas, SS-RMS mainly occurs in infants, children, and adults that similar to ES-RMS and has a median age about 24 years, which affects even younger people than E-RMS. But both E-RMS and SS-RMS mainly arises from the head and neck, and extremity [10, 22–47] (Tables 2 and 3).

**Table 1:** Clinicopathological and prognostic characteristics of epithelioid and spindle rhabdomyosarcoma

Study	Age (Y)/Sex	Size (cm)	Location	Rec/Met	Treatment	Follow-up (Mon)	Outcome
Dashti/2018	70–75/M	4	Mandible	No	No	2	AWND
Wong/2019	20–25/M	8	Nasal cavity	No	Exc, CR	4	DD
Agaram/2019	25–30/F	NA	Skull	NA	NA	NA	NA
Agaram/2019	30–35/F	NA	Maxilla	NA	NA	NA	NA
Agaram/2019	20–25/M	NA	Femur	No	Exc	108	AWND
Agaram/2019	35–40/F	NA	Iliac	NA	NA	NA	NA
Agaram/2019	20–25/F	NA	Femur	L	NA	30	AWD
Tagami/2019	70–75/F	Biopsy	Ver	No	CR	6	AWD
Zhu/2019	70–75/F	4	Maxillary gingiva	NA	NA	NA	NA
Le Loarer/2019	15–20/F	9	Sphenoid	No	Exc, CR	15	DD
Le Loarer/2019	25–30/F	10	Sacrum	L, Med, LN	Ch	4	DD
Le Loarer/2019	35–40/F	NA	Peritoneum	No	Ch	2	DD
Le Loarer/2019	30–35/M	NA	Hard palate & upper lip	Ver & ribs & pelvis	Ch	8	DD
Le Loarer/2019	20–25/M	15	Orbito & temporal & sphenoid	LP	CR	6	DD
Le Loarer/2019	85–90/M	6.5	Inguinal	LP	Exc	6	DD
Le Loarer/2019	15–20/F	5.1	Femur	LP	Exc, Ch	8	DD
Le Loarer/2019	15–20/F	5.3	Cervico-occipital	LP	Exc, Ch	15	AWD
Le Loarer/2019	30–35/M	11.8	Left occipital	L, Med	Exc, Ch	6	DD
Le Loarer/2019	30–35/M	4.5	Mandible	L	Exc, Ch	14	AWD
Le Loarer/2019	55–60/F	1.6	Mandible	No	Exc, CR	21	AWND
Le Loarer/2019	10–15/F	5.5	Mandible	No	Exc, CR	21	AWND
Le Loarer/2019	10–15/F	NA	Maxilla	NA	NA	NA	DD
Le Loarer/2019	25–30/M	3.4	Mandible	No	Exc, Ch	20	AWND
Chrisinger/2020	20–30/F	5	Frontal bone	L	Exc, CR	17	DD
Chrisinger/2020	20–25/F	13.6	Pelvic bones	L, Ver	CR	11	DD
Xu/2021	20–25/M	NA	Mandible	LN	NA	NA	NA
Xu/2021	30–35/M	NA	Mandible	No	NA	10	AWD
Xu/2021	15–20/M	NA	Mandible	L, Bone, LN	Exc, CR	20	DD
Xu/2021	40–45/F	NA	Mandible	NA	NA	NA	NA
Xu/2021	15–20/M	NA	Skull	NA	NA	NA	NA
Xu/2021	25–30/M	NA	Skull	L	NA	2	AWD
Xu/2021	40–45/F	NA	Neck	NA	NA	NA	NA
Our case	**30–35/F**	**5**	**Abdominal wall**	**Med**	**Exc, TCM**	**24**	**AWD**

Rec/Met: Reccurence/Metastasis; L: lung; Med: mediastinal; LN: lymph node; LP: local progression; Ver: Vertebra; Y: years; Exc: Excision; Ch: Chemotherapy; CR: Chemoradiotherapy; TCM: Traditional Chinese Medicine; NA: not available; Mon: months; AWND: alive with no disease; AWD: alive with disease; DD: died of disease

**Table 2:** Clinicopathological and prognostic characteristics of epithelioid rhabdomyosarcoma

Study	Age (Y)/Sex	Size (cm)	Location	Rec/Met	Treatment	Follow-up (Mon)	Outcome
Suarez-Vilela/2004	70–75/M	5.5	Retroauricular & SM	LN	Exc, Ch	3	DD
Fujiwaki/2008	50–55/F	6.5	Fallopian tube	LN	Exc, Ch	6	DD
Bowe/2011	70–75/M	5	Parotid gland LN	No	Exc, Ch	12	AWND
Jo/2011	70–75/M	NA	Knee	L, LN	Exc, CR	10	DD
Jo/2011	70–75/M	5.3	Neck	LN	Exc, Ch	5	DD
Jo/2011	75–80/F	NA	Neck	Med, LN	Rad	2	DD
Jo/2011	30–35/M	8	Arm	L, bone	Exc, CR	60	DD
Jo/2011	20–25/M	8.5	Thigh	L, Liver	Exc, Ch	6	DD
Jo/2011	10–15/F	NA	Elbow	No	Exc, CR	47	AWND
Jo/2011	35–40/M	8	Forearm	L, LN	Exc, CR	10	DD
Jo/2011	75–80/M	8	Shoulder	L	Exc	24	DD
Jo/2011	70–75/M	5	Chesk wall	No	Exc, Ch	36	AWND
Zin/2014	5–10/M	NA	Para-meningeal	LN	Exc, Ch	96	AWND
Zin/2014	5–10/M	3.3	Para-meningeal	NA	Exc, Ch	120	AWND
Zin/2014	10–15/F	4	Arm	No	Exc, Ch	72	AWND
Zin/2014	5–10/F	8.3	Arm	LN	Exc, Ch	24	AWND
Zin/2014	5–10/M	4	Orbit	No	Exc, Ch	48	AWND
Yu/2015	15–20/F	15	Left thigh	NA	NA	4	DD
Yu/2015	75–80/M	12	Left waist & back	Rec	Exc, Rad	13	AWND
Yu/2015	60–65/M	12.5	Left chesk wall	LN	Exc	2	AWND
Yu/2015	55–60/M	2.5	Left femur	L	Exc, Ch	14	DD
Yu/2015	80–85/F	NA	Left upper eyelid	Preaurical	Exc	7	DD
Yu/2015	35–40/M	3.5	Thyroid gland	No	Exc, CR	6	AWND
Jokoji/2015	65–70/F		Neck & abdomen	LN	Ch	6	DD
Renshaw/2019	50–55/F	NA	Pleural & L & RP	NA	NA	21(Days)	DD
Valerio/2020	80–85/M	3	Neck cutaneous	NA	NA	0	DD
De Aguiar/2020	15–20/M	6	Jaw	NA	Exc, Ch	5	DD
Rodgers/2021	70–75/M	11.5	Pelvic	LN	No	5(Days)	DD

Rec/Met: Reccurence/Metastasis; L: lung; Med: mediastinal; LN: lymph node; LP: local progression; Ver: Vertebra; SM: submandibular; Y: years; Exc: Excision; Ch: Chemotherapy; CR: Chemoradiotherapy; Rad: Radiotherapy; TCM: Traditional Chinese Medicine; NA: not available; Mon: months; AWND: alive with no disease; AWD: alive with disease; DD: died of disease

**Table 3:** Clinicopathological and prognostic characteristics of spindle cell/sclerosing rhabdomyosarcoma

Study	Age (Y)/Sex	Size (cm)	Location	Rec/Met	Treatment	Follow-up (Mon)	Outcome
Mentzel/2006	35–40/M	13	Forearm	L	Exc	24	DD
Mentzel/2006	55–60/M	19	Hip	No	Exc	46	AWND
Mentzel/2006	60–65/M	11	Lower leg	L	Exc	12	DD
Mentzel/2006	75–80/F	4	Thigh	No	Exc	15	AWND
Mentzel/2006	50–55/M	8	Neck	No	Exc, Ch	48	AWND
Gavino/2010	30–35/F	NA	Right leg	Met	Exc	16	AWD
Rekhi/2014	15–20/M	NA	Oral cavity and maxilla	No	Exc, CR	12	AWD
Rekhi/2014	15–20/M	NA	Paratesticular	LN	Exc	12	AWND
Rekhi/2014	60–65/M	NA	Thigh	L	Exc, Ch	5	AWD
Rekhi/2014	30–35/F	NA	Cheeck	NA	CR	16	DD
Rekhi/2014	35–40/M	NA	Paraspinal	Lung	Exc	2	AWD
Mikubo/2014	25–30/F	13	Chest wall	Rec	Exc, Ch	18	AWD
Yasui/2015	20–25/F	> 5	Pharynx	LN	Exc, CR	27.1	DD
Yasui/2015	25–30/F	> 5	Malar region	Rec & Met	Exc, Rad	74.6	DD
Yasui/2015	35–40/M	> 5	Dorsum of the foot	No	Exc	144.7	AWND
Yasui/2015	15–20/M	> 5	Temporal region	Rec & Met	Exc, CR	24.3	DD
Yasui/2015	45–50/M	< 5	Scrotum	Rec & Met	Exc, Ch	14.2	DD
Yasui/2015	10–15/M	> 5	Scrotum	No	Exc, Ch	201	AWND
Yasui/2015	20–25/F	> 5	Malar region	No	Exc, CR	39.9	AWND
Yasui/2015	25–30/F	> 5	Intrathoracic space, thoracic wall	Rec	Exc, CR	40.6	DD
Yasui/2015	15–20/F	< 5	Malar region, pharynx	Rec & Met	CR	116.8	DD
Yasui/2015	15–20/M	> 5	Parapharyngeal space	Rec & Met	Exc, CR	24.7	DD
Yasui/2015	20–25/F	< 5	Tongue	Rec & Met	Exc, Ch	38.4	DD
Yasui/2015	35–40/M	> 5	Prostate	NA	Exc, Ch	89	AWND
Yasui/2015	25–30/M	> 5	Malar region	NA	Exc, CR	14.6	AWND
Yasui/2015	5–10/M	> 5	Parapharyngeal space	Rec	Exc, CR	14.4	AWND
Yasui/2015	20–25/M	> 5	Scrotum	NA	Exc, Ch	4.7	AWD
Zhao/2015	40–45/M	5.2	Left upper arm	NA	Exc, CR	24	DD
Zhao/2015	30–35/M	14	Abdomen wall	Rec	Exc	10	AWD
Zhao/2015	45–50/M	2.4	Larynx	No	Exc, Rad	5	AWND
Zhao/2015	0–5/F	2.8	Left orbit	NA	Exc	5	AWD
Zhao/2015	25–30/M	5	Nasopharynx	NA	Exc	1	AWD
Zhao/2015	50–55/M	21	Left thigh	Rec & Met	Exc	12	AWD
Zhao/2015	25–30/F	3.4	Back of right hand	No	Exc	1	AWND
Zhao/2015	15–20/M	16.5	Right groin	No	Exc, Ch	6	AWND
Zhao/2015	35–40/F	4.8	Left pars buccalis	No	Exc	8	AWND
Zhao/2015	55–60/F	2.3	Right parotid gland	No	Exc	13	AWND
Alaggio/2016	0–5/F	NA	Back	NA	NA	108	AWND
Alaggio/2016	0–5/F	NA	Back	NA	NA	72	AWND
Alaggio/2016	0–5/F	NA	Lower neck/back	NA	NA	96	AWND
Alaggio/2016	15–20/M	NA	Paravertebral	NA	NA	24	DD
Alaggio/2016	10–15/F	NA	Buttock	NA	NA	6	DD
Alaggio/2016	5–10/M	NA	Thigh	NA	NA	1	AWND
Alaggio/2016	5–10/M	NA	Head and Neck	Rec	NA	36	AWD
Alaggio/2016	5–10/F	NA	Head and Neck	NA	NA	24	DD
Alaggio/2016	0–5/M	NA	Intra-abdomina	NA	NA	156	AWND
Alaggio/2016	15–20/M		Paratesticular			24	AWND
Alaggio/2016	0–5/F	NA	Ovary/salpinx	NA	NA	48	DD
Alaggio/2016	10–15/M	NA	Paratesticular	NA	NA	24	AWND
Walther/2016	0–5/F	5	Thigh	NA	Exc, Ch	16	DD
Owosho/2016	15–20/F	3.5	Buccal/masticator space	NA	Exc, CR	31	AWND
Owosho/2016	25–30/M	10.5	Buccal/masticator space	NA	CR	12	DD
Owosho/2016	30–35/M	5.8	Soft tissue mandible	Rec & Met	Exc, CR	65	DD
Owosho/2016	70–75/M	9.1	Neck	NA	Exc, CR	29	AWND
Owosho/2016	40–45/M	4.5	Soft tissue mandible	NA	Exc, Rad	4	AWND
Owosho/2016	60–65/M	8.3	Hypopharynx	NA	Exc, CR	14	DD
Owosho/2016	30–35/M	0.8	Tongue	NA	Exc, Ch	94	AWND
Owosho/2016	0–5/F	1	Nasolabial/cheek	NA	Exc, Rad	7	AWND
Smith/2017	20–25/M	5.1	Oral cavity	No	Exc, CR	48	AWND
Momosaka/2017	65–70/F	NA	Intracranial	Rec	Exc	3	DD
Agaram/2019	10–15/F	NA	Head and Neck	Rec & Met	CR	48	AWND
Agaram/2019	35–40/M	NA	Lower leg	NA	CR	60	AWND
Agaram/2019	75–80/M	NA	Lower leg	Rec & Met	CR	32	DD
Whittle/2019	0–5/M	2.7	Palpable	No	Exc, Ch	15	AWND
Whittle/2019	0–5/F	5.2	Chest wall	No	Exc, Ch	12	AWND
Whittle/2019	0–5/F	NA	Chest wall	No	Exc, Ch	13	AWND
Whittle/2019	0–5/F	4.2	Left foot	No	Exc, Ch	7	AWND
Akki/2019	55–60/F	18.5	Liver	No	Ch	12	AWND
Cordier/2021	0–5/M	4.8	Below the left scapula	No	Ch	6	AWD
Jariod-Ferrer/2021	25–30/M	0.9	Tongue	Rec	Exc, CR	48	AWD
Wang/2021	55–60/M	7	Left temporal scalp	No	Exc, CR	3	AWND

Rec/Met: Reccurence/Metastasis; L: lung; Med: mediastinal; LN: lymph node; LP: local progression; Ver: Vertebra; Y: years; Exc: Excision; Ch: Chemotherapy; CR: Chemoradiotherapy; Rad: Radiotherapy; TCM: Traditional Chinese Medicine; NA: not available; Mon: months; AWND: alive with no disease; AWD: alive with disease; DD: died of disease

**Fig. 5:**
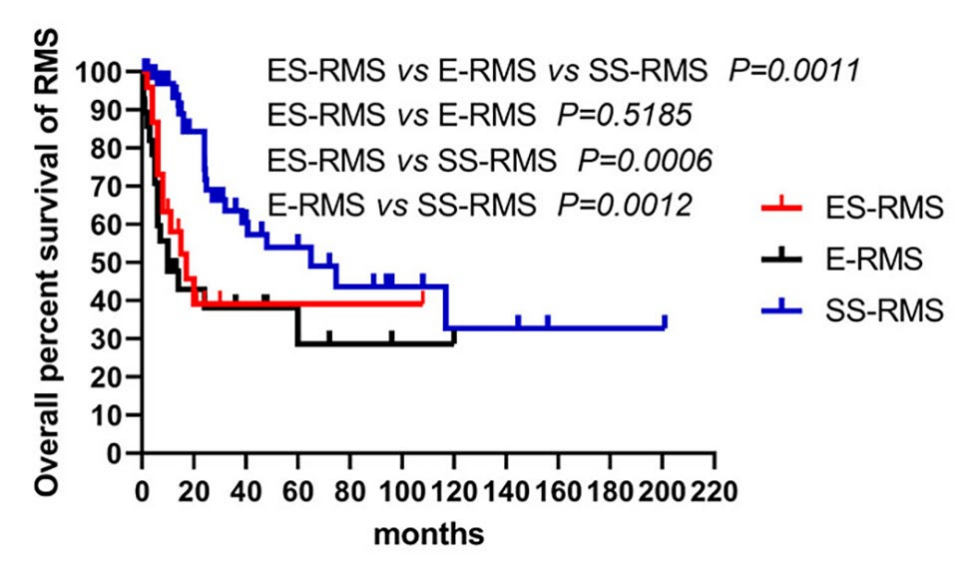
Prognostic comparisons of RMS. ES-RMS: epithelioid and spindle rhabdomyosarcoma; E-RMS: epithelioid rhabdomyosarcoma; SS-RMS: spindle cell/sclerosing rhabdomyosarcoma

Additionally, we found that many cases of ES-RMS had local progression or metastasis, and up to 50% (12/24) of ES-RMS had died of disease within the follow-up times, similar to E-RMS (median survival time is 10 month) [22–32], ES-RMS showed a more aggressive behavior and adverse prognosis (median survival time is 17 month) than SS-RMS (median survival time is 65 month) [10, 12, 15–17, 19–21, 33, 34, 36–40, 42–47] (Tables 1, 2 and 3; Fig. 5).

## Discussion

ES-RMS is a rare neoplasm characteristic by mainly harboring EWSR1-TFCP2 or FUS-TFCP2 fusion, which is mainly distracted from SS-RMS and distinctive from other variants of RMS, such as pleomorphic, alveolar, spindle cell/sclerosing, embryonal RMS. The neoplasm was firstly descripted with RNA sequencing by Watson and colleagues in 2018 [48]. Later, merely 32 cases were documented in English literature and clinicopathological features were good summarized in the latest paper as well [12, 15].

It is not uncommon that neoplasm cells of ES-RMS were immunoreactivity for ALK protein according to the previous studies [15, 21], so was confirmed in our present case, which is rarely seen in the other variants of RMS. Therefore, it strongly indicates that co-expression skeletal muscle markers and ALK may be a distinct phenomenon in ES-RMS. However, until now, no case of ES-RMS including our present case has found ALK re-arrangement at genetic levels, but ALK deletion in exons or introns were detected in the four cases with FUS-TFCP2 rather than EWSR1-TFCP2 fusion [17, 21]. It seems that alterative underlying mechanisms may involve in ALK over-expression, which needs to be further unmasked in the future.

Unexpectedly, when we further detected whether the neoplasm cells of ES-RMS exist ROS1 mutation, we firstly found tumor cells showed not only ROS1 mutation but also increased copy numbers. To date, no increased copy numbers of ROS1 gene had reported in English literature in human tumors including cancer and mesenchymal tumor, nor ROS1 re-arrangement in RMS including ES-RMS. On the contrary, a variety of ROS1 re-arrangement or mutation were detected in various tumors, such as ROS1-EPHA7 fusion in breast cancer [49], ROS1-FN1 or ROS1-SLC12A2 re-arrangement in inflammatory myofibroblastic tumor [50, 51], ROS1-WNK1 or ROS1-CD74 fusion or ROS1-G2032R mutation in lung cancer [52–54]. Some of these fusions or mutations showed, to some degree, clinical significance, for instance, like ALK positive non-small cell lung cancer (NSCLC) [55], ROS1 positive NSCLC has more high risk of central nervous system metastasis [56]. However, we don’t know what’s the significance of increased copy numbers of ROS1 gene in the ES-RMS, it seemly implying that ROS1 gene mutation or increased copy numbers may play a crucial role in the malignant progression, thereby, it will be necessary to unmask the significance of increased copy numbers of ROS1 gene in the ES-RMS, if very fortunate, we may find molecular drugs precisely targeting ROS1, thus opening a promising window for effective and safe treatment on ES-RMS in the future.

EWSR1 re-arrangement was found in a variety of tumors, such as EWSR1-NFATC2 re-arrangement in both simple bone cyst and vascular malformation/hemangioma [57], EWSR1-ATF1 fusion in pediatric mesothelioma [58], EWSR1-CREB3L1 fusion in sclerosing epithelioid fibrosarcoma [59], EWSR1 amplification in clear cell myoepithelial carcinomas [60]. Unexpectedly, we detected increased copy numbers of EWSR1 locus in many neoplasm cells of ES-RMS, which is a more intriguing phenomenon, as until now, no increased copy numbers of EWSR1 gene were identified in any case in the English literature. Similar to increased copy numbers of ROS1 gene, we know nothing about significance of increased copy numbers of EWSR1 gene in the ES-RMS as well, but find a combination of increased copy numbers of ROS1 and EWSR1 gene, along with high TMB in neoplasm cells, we can reasonably speculate that increased copy numbers of ROS1 and EWSR1, to some extends, may play synergistic functions on the malignant aggression on the background of high TMB in neoplasm cells.

Although the next-generation sequencing was performed, the partner gene of TFCP2 was not identified regretfully, which may be partially attributed to the reason that the TFCP2 gene and its partner gene were not comprised in the panel of sequencing genes, but based on the morphological, immunohistochemical features in combination with the published articles, we strongly convinced that a consideration of ES-RMS should not be dropped. Therefore, we employed FISH to determine whether the tumor harbors TFCP2 rearrangement or not. Fortunately, although the partner gene of TFCP2 was not identified, TFCP2 rearrangement was finally confirmed, indicating that a small subset of ES-RMS had TFCP2 with other unknown gene fusions or alterations.

Previous research confirmed that MET mutation may be contribute to the malignant progression, distant metastasis and poor differentiation of RMS cells [61, 62], we found that ES-RMS in our case frequently displays MET gene mutation as well as increased copy numbers, it seems that MET mutation may, to a certain extent, be contribute to the malignant behavior.

Moreover, the latest study had confirmed that the RMS patients without gene fusion easily display more frequent genomic mutations, a higher TMB and poorer prognosis than those with gene fusion [14], and high TMB may be a predictive biomarker for pembrolizumab, a monoclonal antibody drug anti-PD-1 in human solid tumor [63], in keeping with this, the patient in our study showed TFCP2 rearrangement and had frequent genetic alteration and high TMB, it indicates that a small subset of ES-RMS with TFCP2 rearrangement had high TMB and increased copy numbers of a variety of genes, seeming that these patients with high TMB may be benefit from the immune checkpoint inhibitors.

The main differential diagnostic consideration includes inflammatory myofibroblastic tumor (IMT), nodular fasciitis, proliferative fasciitis and myositis, squamous cell carcinoma, epithelioid sarcoma, malignant melanoma, and other variants of RMS. IMT is the tumor that most frequently overexpresses ALK protein, however, in addition to no expression of skeletal muscle markers Myo-D1, Myogenin and Myogenin, genetically, IMT usually shows ALK re-arrangement with many other molecular partners including TPM3, KIF5B, CARS, and THBS1 [51]. Nodular fasciitis is a benign lesion classically arising in the young adults and displays myofibroblast in tissue culture within variable myxoid or collagen-rich stroma, cystic and hemorrhagic space, and a loose storiform growth pattern, along with scattered lymphocytes, plasma cells, osteoclast-like giant cells, it strongly expresses smooth muscle actin but usually neither desmin nor Myo-D1 and Myogenin, more importantly, it is characterized by harboring MYH9-USP6 fusion that lacked in ES-RMS [64, 65]. Proliferative fasciitis and myositis is the lesions that composed of ganglion-like fibroblast cells that seen in the background of nodular fasciitis-like appearance or checkerboard-like appearance with degenerating skeletal muscle that alarming RMS, however, these lesions may express actin and some histiocytic markers and lack expression of desmin, Myo-D1 and Myogenin [66, 67], in addition, a small subset of cases can characteristically express c-FOS protein and genetically harbor FOS gene re-arrangement [68]. Squamous cell carcinoma can, to some extents, shows epithelioid and spindle cells but generally exists carcinoma in situ at peripheral surface epithelium, although rare cases express Myogenin protein, but they usually display strongly positive for CK5/6, p63 and p40, and are negative for Desmin and Myo-D1 [69]. Epithelioid sarcoma can show rhabdoid and spindle morphology with tumor necrosis, they generally express epithelial markers and CD34, and loss SMARCB1/INII1 expression and don’t express Myo-D1 and Myogenin [70, 71]. Rare malignant melanoma can show RMS dedifferentiation, however, it usually presents previous history of melanoma and melanoma in situ and is diffusely positive for S-100, SOX10, HMB45 and Melan A in the classic components of melanoma cells, molecularly, BRAF VE600 mutation can be detected in some melanoma [72, 73]. Other variants of RMS including ES-RMS, SS-RMS can be separated by the combination of cellular components and molecular testing in the appropriate clinical background.

Due to the poor prognosis of ES-RMS analogous to E-RMS compared with SS-RMS, it is reasonable to speculate that, morphologically, emerging of epithelioid cell component may be a bad factor for RMS and indicates a more aggressive behavior, it is therefore important to differentiate ES-RMS from SS-RMS and precise targeted therapy.

A great numbers of ES-RMS can detect ALK over-expression but no ALK re-arrangement was found, targeting ALK may be a potential therapeutic tool, up to now, it fails to obtain satisfactory therapeutic efficacy on the RMS [74, 75]. In addition, drugs targeting ROS1 re-arrangement have already approved and obtained favorable effects in NSCLC patients [76], although ROS1 mutation and increased copy numbers were detected in ES-RMS, but no ROS1 re-arrangement was detected, therefore, we still don’t know whether targeting ROS1 mutation or increased copy numbers will be valuable. Moreover, frequent MET mutation in ES-RMS may as well open another window for potential novel targeting therapy, recent study has indicated that crizotinib may abrogate RMS cells proliferation, viability, migration and invasion by inhibiting both ALK, MET and Insulin-like growth factor 1 receptor to induce cells autophagy and apoptosis [77]. Moreover, high TMB indicates the patients may be benefit from the immune checkpoint inhibitors [63]. In brief, some novel drugs targeting ALK, ROS1, MET or immune checkpoint inhibitors may be potential and promising tools for the treatments of ES-RMS in the future.

## Conclusions

As ES-RMS is rare, high malignancy and easy to misdiagnose as other entities, we report just mere one case of ES-RMS, and unexpectedly, we can’t confirm EWSR1 or FUS fusion in this case, indicating that a small subset of ES-RMS had TFCP2 with other unknown gene fusions or alterations, such as MET mutation, increased copy numbers of EWSR1 and ROS1 gene, high TMB. We hope more cases and studies will unmask molecular genetic characteristic of ES-RMS in the future.

## Electronic supplementary material

Below is the link to the electronic supplementary material.

**Supplementary Fig. 1** Color Doppler Ultrasound findings: Color Doppler Ultrasound showed a hypoechoic nodule about 0.57 × 0.52 × 0.64 cm in the subcutaneous soft tissue of the right abdominal wall

**Supplementary Table 1** MET gene exon14 mutation in chromosome7 by next-generation sequencing. Ref: reference; Alt: alteration; freq: frequency; SNV: single nucleotide variation

**Supplementary Table 2** 15 somatically acquired gene mutations detected by next-generation sequencing. chr: chromosome; Ref: reference; Alt: alteration; freq: frequency; SNV: single nucleotide variation

## Data Availability

Not applicable.
