# Supplementary figures and images for: Epithelioid and spindle rhabdomyosarcoma with TFCP2 rearrangement in abdominal wall: a distinctive entity with poor prognosis
Source: Diagn Pathol. 2023 Mar 30;18:41. doi: 10.1186/s13000-023-01330-y (PMC10061849; doi:10.1186/s13000-023-01330-y)

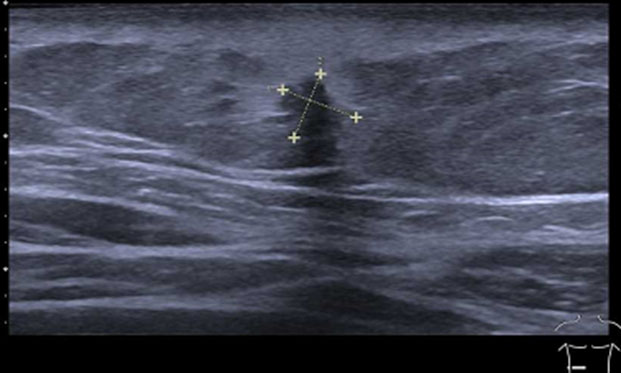

Supplement: Supplementary file 1 — Supplementary Fig. 1 Color Doppler Ultrasound findings: Color Doppler Ultrasound showed a hypoechoic nodule about 0.57 × 0.52 × 0.64 cm in the subcutaneous soft tissue of the right abdominal wall [file 13000_2023_1330_MOESM1_ESM.jpg]
