# Supplementary material for: Epithelioid and spindle rhabdomyosarcoma with TFCP2 rearrangement in abdominal wall: a distinctive entity with poor prognosis
Source: Diagn Pathol. 2023 Mar 30;18:41. doi: 10.1186/s13000-023-01330-y (PMC10061849; doi:10.1186/s13000-023-01330-y)
Supplement: Supplementary file 2 — Supplementary Table 1 MET gene exon14 mutation in chromosome7 by next-generation sequencing. Ref: reference; Alt: alteration; freq: frequency; SNV: single nucleotide variation [file 13000_2023_1330_MOESM2_ESM.docx]

**Supplementary table 1** MET gene exon14 mutation in chromosome7 by next-generation sequencing.

| Start | End | Ref | Alt | freq | hgvs_cds | hgvs_protein | ExonicFunc.refGene |
| --- | --- | --- | --- | --- | --- | --- | --- |
| 116771869 | 116771869 | C | T | 0.0029 | c.C2908T | p.R970C | nonsynonymous SNV |
| 116771888 | 116771888 | A | G | 0.0018 | c.A2927G | p.H976R | nonsynonymous SNV |
| 116771891 | 116771891 | C | T | 0.0027 | c.C2930T | p.T977I | nonsynonymous SNV |
| 116771894 | 116771894 | C | T | 0.0036 | c.C2933T | p.P978L | nonsynonymous SNV |
| 116771912 | 116771912 | T | A | 0.0019 | c.T2951A | p.V984E | nonsynonymous SNV |
| 116771920 | 116771920 | C | T | 0.002 | c.C2959T | p.R987X | stopgain |
| 116771931 | 116771931 | C | T | 0.002 | c.C2970T | p.S990S | synonymous SNV |
| 116771936 | 116771936 | C | T | 0.0041 | c.C2975T | p.T992I | nonsynonymous SNV |
| 116771939 | 116771939 | C | T | 0.0022 | c.C2978T | p.T993I | nonsynonymous SNV |
| 116771950 | 116771950 | T | - | 0.0023 | c.2989delT | p.S997Qfs*5 | frameshift deletion |

Ref: reference; Alt: alteration; freq: frequency; SNV: single nucleotide variation.
