# Supplementary material for: Epithelioid and spindle rhabdomyosarcoma with TFCP2 rearrangement in abdominal wall: a distinctive entity with poor prognosis
Source: Diagn Pathol. 2023 Mar 30;18:41. doi: 10.1186/s13000-023-01330-y (PMC10061849; doi:10.1186/s13000-023-01330-y)
Supplement: Supplementary file 3 — Supplementary Table 2 15 somatically acquired gene mutations detected by next-generation sequencing. chr: chromosome; Ref: reference; Alt: alteration; freq: frequency; SNV: single nucleotide variation [file 13000_2023_1330_MOESM3_ESM.docx]

**Supplementary table 2** 15 somatically acquired gene mutations detected by next-generation sequencing.

| Chr | Start | End | Ref | Alt | freq | gene | hgvs_cds | hgvs_protein | exon_id | ExonicFunc.refGene |
| --- | --- | --- | --- | --- | --- | --- | --- | --- | --- | --- |
| chr6 | 117301068 | 117301068 | G | T | 0.575 | ROS1 | c.C6639A | p.D2213E | exon42 | nonsynonymous SNV |
| chr5 | 233627 | 233627 | T | G | 0.559 | SDHA | c.T1046G | p.L349R | exon8 | nonsynonymous SNV |
| chr8 | 37841925 | 37841925 | G | A | 0.537 | ADGRA2 | c.G3587A | p.C1196Y | exon19 | nonsynonymous SNV |
| chr2 | 136115243 | 136115243 | A | T | 0.504 | CXCR4 | c.T685A | p.S229T | exon2 | nonsynonymous SNV |
| chr2 | 140456486 | 140456486 | C | G | 0.485 | LRP1B | c.G9932C | p.R3311T | exon62 | nonsynonymous SNV |
| chr14 | 103698932 | 103698932 | C | T | 0.47 | XRCC3 | c.G907A | p.E303K | exon10 | nonsynonymous SNV |
| chr2 | 215338894 | 215338894 | C | G | 0.454 | ATIC | c.C1214G | p.T405S | exon12 | nonsynonymous SNV |
| chr1 | 204429933 | 204429936 | AGGG | GGGA | 0.382 | PIK3C2B | c.4383_4386delinsTCCC | p.P1461P | exon30 | nonframeshift substitution |
| chr4 | 1976628 | 1976628 | T | C | 0.235 | NSD2 | c.T3775C | p.C1259R | exon21 | nonsynonymous SNV |
| chr1 | 158667939 | 158667939 | A | G | 0.153 | SPTA1 | c.T1957C | p.Y653H | exon15 | nonsynonymous SNV |
| chr2 | 189854896 | 189854899 | AATG | CATT | 0.128 | PMS1 | c.1624_1627delinsCATT | p.N542T | exon9 | stopgain |
| chr20 | 32434459 | 32434459 | T | A | 0.117 | ASXL1 | c.T1747A | p.W583R | exon12 | nonsynonymous SNV |
| chr19 | 29820718 | 29820718 | A | G | 0.109 | CCNE1 | c.A479G | p.K160R | exon7 | nonsynonymous SNV |
| chrX | 47567267 | 47567267 | A | T | 0.108 | ARAF | c.A911T | p.E304V | exon10 | nonsynonymous SNV |
| chr20 | 41115374 | 41115374 | T | C | 0.104 | TOP1 | c.T1642C | p.F548L | exon16 | nonsynonymous SNV |
| chr16 | 81891492 | 81891495 | ACGA | TCGT | 0.104 | PLCG2 | c.888_891delinsTCGT | p.S296S | exon11 | nonframeshift substitution |

chr: chromosome; Ref: reference; Alt: alteration; freq: frequency; SNV: single nucleotide variation.
